# Behind and Beyond the Masaoka Staging: A 25-Year Follow-up Study of Tumor Recurrence in Completely Resected Thymic Epithelial Tumors in a Single Institution

**DOI:** 10.1097/MD.0000000000002278

**Published:** 2015-12-31

**Authors:** Yau-Lin Tseng, Jia-Ming Chang, Wu-Wei Lai, Kung-Chao Chang, Shang-Chi Lee, Sheng-Hsiang Lin, Yi-Ting Yen

**Affiliations:** From the Division of Thoracic Surgery (Y-LT, W-WL, Y-TY), Department of Surgery, National Cheng Kung University Hospital, College of Medical College, National Cheng Kung University, Tainan; Division of Thoracic Surgery (J-MC), Department of Surgery, Chia-Yi Christian Hospital, Chia-Yi; Department of Pathology (K-CC), National Cheng Kung University Hospital, College of Medical College, National Cheng Kung University; Biostatistics Consulting Center (S-CL), National Cheng Kung University Hospital, College of Medical College, National Cheng Kung University; and Institute of Clinical Medicine (S-HL, Y-TY), National Cheng Kung University, Tainan, Taiwan.

## Abstract

We analyzed prognosticators for recurrence and post-recurrence survival in completely resected thymic epithelial tumors for the past 25 years in a single institution.

Between June 1988 and December 2013, 238 patients undergoing intent-to-treat surgery for thymic epithelial tumors were reviewed. Sex, age, myasthenia gravis (MG), tumor histology, Masaoka staging, characteristic of locoregional invasion and recurrence, and the treatment for recurrence were collected. Comparison between groups was conducted using the Student *t* test and χ^2^ test. Survival analysis was performed using the Kaplan–Meier method and log-rank test. The Cox proportional hazards model was used for univariate and multivariate analyses of prognostic factors.

One hundred sixteen of 135 patients with completely resected thymoma and 35 of 56 patients with thymic carcinoma remained free of recurrence. In patients with completely resected thymoma, Masaoka staging, MG, tumor invasion into the lung, pericardium, and innominate vein or superior vena cava (SVC) invasion were associated with recurrence-free survival in univariate analysis (*P* = 0.004, 0.003, 0.001, 0.007, and 0.039, respectively). In multivariate analysis, MG was the positive independent prognosticator (*P* = 0.039). In patients with completely resected thymic carcinoma, Masaoka staging and innominate vein or SVC invasion were associated with recurrence-free survival in univariate analysis (*P* = 0.045 and 0.005, respectively), whereas innominate vein or SVC invasion was the negative independent prognosticator (*P* = 0.012). In patients with recurrent thymoma, those treated with surgery followed by chemotherapy had a significantly better post-recurrence survival than those undergoing chemoradiotherapy (*P* = 0.029) and those without treatment (*P* = 0.007). Patients with recurrent thymic carcinoma undergoing surgery followed by chemotherapy had a significantly better post-recurrence survival than those without treatment (*P* = 0.004), but not significantly better than those undergoing chemoradiotherapy (*P* = 0.252).

In patients with completely resected thymoma, MG was the positive independent prognosticators of recurrence-free survival. Surgery should be attempted for recurrent disease for better post-recurrence survival. In patients with completely resected thymic carcinoma, innominate vein or SVC invasion was the negative independent prognosticator. Surgery for recurrence could be considered since it provided benefit for post-recurrence survival as chemoradiotherapy did.

## INTRODUCTION

Thymic epithelial tumors (TETs) consist of thymoma and thymic carcinoma, and they tend to be treated fairly similarly despite the fact that the update of the World Health Organization classification in 2004 officially recognized the distinction between thymic carcinoma and thymoma as separate and distinct histologic entities.^1^ Although complete resection provides the best survival and opportunity of cure, approximately 10% to 30% of patients with TETs undergoing surgical resection developed recurrent diseases.^2–4^ Factors predicting recurrence and the optimal treatment for recurrent disease have been investigated; however, patients underwent incomplete resection were included.^2,3,5–7^ Patients with TETs have a number of characteristics which make overall survival problematic for assessment of tumor-related outcomes. More specific measures are mandatory in addition to overall survival. Given that long-term survival is achievable with resection and recurrence may provide a more clinically relevant end point,^8,9^ the present study retrospectively analyzes the prognostic factor in patients with completely resected thymoma and thymic carcinoma. Prognostic statements and treatment strategies for recurrence in TETs can therefore be made on the basis of greater patient collectives of those undergoing complete resection and longer follow-up.

## MATERIALS AND METHODS

### Patient Enrollment

A retrospective review of medical records between June 1988 and December 2013 was conducted among 238 patients who underwent intent-to-treat surgery for TET. Informed consent was waived because the study was retrospective, and the review of medical records was approved by the Institutional Review Board of National Cheng Kung University Hospital (A-ER-103–189). The surgical pathologies, including the tumor histology and resection margin, were microscopically confirmed by an experienced pathologist, who was blind to the clinical data, and the tumors were classified according to the newly published World Health Organization classification. Clinical information was collected on sex, age, myasthenia gravis, tumor histology, Masaoka staging, characteristics of locoregional invasion, sites of recurrence, and the treatment modalities for recurrent disease. Tumor histology, Masaoka stage, and characteristics of tumor invasion were included in the analysis of prognostic factors for recurrence-free survival.

### General Management Principles for TETs

Computed tomography or magnetic resonance imaging was used to evaluate whether the lesions were resectable. Abdominal sonography and whole-body bone scan were routine preoperative metastatic evaluation modalities. Biopsy was performed by mediastinotomy, mediastinoscopy, or thoracoscopy for unresectable lesions. Preoperative cisplatin-based chemotherapy along with radiation therapy (3000–5000 cGy) were administered to patients whose imaging studies showed locally advanced and unresectable diseases, including superior vena cava syndrome, pericardial effusion with encasement of the pulmonary vessels or aorta. The chemotherapy consisted of 3 cycles of cisplatin (60 mg/m^2^, day 1) and etoposide (120 mg/m^2^, day 1) every 3 weeks. Postoperative chemotherapy was given to patients who had residual or recurrent disease. The regimen was composed of 3 cycles of cisplatin (50 mg/m^2^, day 1), doxorubicin (50 mg/m^2^, day 1), and cyclophosphamide (500 mg/m^2^, day 1) every 3 weeks. Operative procedures were subsequently performed if the tumor had been downstaged radiographically and evaluated as resectable. Postoperative radiation therapy with a full dose (5000–6000 cGy) was routinely performed at the tumor bed and mediastinum for patients with advanced diseases undergoing complete resection if they were not preoperatively irradiated. For those with residual, recurrent disease or previously irradiated, the dose of radiotherapy was tailored accordingly so that it was limited to a total dose of 6000 cGy in the mediastinum.

### Surgery for TET

Radical resection, including extended thymectomy and thymomectomy, was the surgical principle. The phrenic or recurrent laryngeal nerve was preserved on condition that the tumor capsule was not violated. Resection of the nerve was otherwise performed if it was severely adhered or encased by the tumor. The lung parenchyma was resected using surgical staples instead of dissection if adhesion between the tumor and lung parenchyma was identified. For tumors involving more than half the circumference of SVC, we did total excision followed by prostheses (ringed GoreTex, woven Dacron and pericardial tube graft) interposition as in our previous report.^10^ Direct resection and repair were done if less than half of the circumference was involved and the blood flow was not compromised; patched repair with equine pericardium was done instead, if the lumen would have been compromised.

### Follow-up and Evaluation of Recurrence

All the patients were followed up for at least 6 months or until the time of their death. For patients with clinical appearance of new diseases on follow-up imaging after a complete resection, thoracoscopy was the option of diagnostic and therapeutic modality for intrathoracic lesions. Extrathoracic lesions were determined by biopsy and tissue proof whenever possible or the consensus of a multidisciplinary panel discussion based on the Response Evaluation Criteria in Solid Tumors guideline.^11,12^ Recurrence was divided into 3 categories according to the definition of the International Thymic Malignancy Interest Group.^9^ Recurrent disease was treated with surgery followed by chemotherapy if it was localized and with chemoradiation therapy if not surgically feasible. Patients who died of cancers other than TETs were defined to have non-thymic tumor-related death, whereas mortality resulted from adverse effect or complication of treatment was defined as treatment-related death. The survival period after recurrence was computed from the date of recurrence diagnosis to the date of the last follow-up or death.^9^

### Statistical Analysis

Continuous variables between groups were compared using the Student *t* test, while categorical variables were compared using the χ^2^ test. When the numbers in a cell of comparison table were smaller than 5, Fisher exact test was used for comparison between 2 groups, and Spearman rank correlation test for comparison among 3 groups. Survival analysis was performed using the Kaplan–Meier method, and the statistical difference was determined using the log-rank test. Cox proportional hazards model was used for univariate and multivariate analyses of prognostic factors, and test for multicollinearity using Pearson correlation was also conducted. Statistical significance was set at *P* < 0.05. All statistical analysis was performed using SPSS version 20.0 for Windows (IBM Corp, Armonk, NY).

## RESULTS

A total number of 238 patients with TET underwent intent-to-treat surgery. One hundred ninety-one of the 238 patients had microscopically complete (R0) resection, 13 had microscopically incomplete (R1) resection, and 34 had macroscopically incomplete (R2) resection (Table 1). There were 3 patients with stage IV thymoma and 8 patients with stage IV thymic carcinoma undergoing complete resection because they had pathologically confirmed regional lymph node or solitary pulmonary metastasis, instead of pleural or pericardial metastasis, which were radically resectable. Since significant differences were noted in histology (*P* = 0.002) and Masaoka stage (*P* = 0.000) among patients undergoing R0, R1, and R2 resection, further comparison between each group was performed (Table 2). While significant difference in histology existed only between patients undergoing R0 and R2 resection (*P* = 0.000), the differences in Masaoka stage were all significant between patients undergoing R0 and R1resection (*P* = 0.002), R0 and R2 resection (*P* = 0.000), and R1 and R2 resection (*P* = 0.000). Among those 191 patients undergoing complete resection for TETs, 19 of the 134 with completely resected thymoma and 21 of the 56 patients with completely resected thymic carcinoma had recurrent diseases (Table 3). The proportion of recurrent disease was significantly associated with Masaoka staging, lung invasion, pericardial invasion, innominate vein or superior vena cava (SVC) invasion, and lymph node metastasis in patients with completely resected thymoma (*P* = 0.036, 0.008, 0.007, 0.049, and 0.036, respectively). In patients with completely resected thymic carcinoma, the proportion of recurrent disease was significantly associated with innominate vein or SVC invasion, and preoperative radiotherapy (*P* = 0.002 and 0.016, respectively). Nonetheless, the evaluation of prognosticators for recurrence after complete resection relies on the recurrence-free survival because tumor recurrence is a time-dependent event. In patients with completely resected thymoma, the results of univariate analysis revealed that Masaoka staging, myasthenia gravis, lung invasion, pericardium invasion, and innominate vein or SVC invasion were significantly associated with recurrence-free survival (*P* = 0.004, 0.003, 0.001, 0.007, and 0.039, respectively; Table 4). In multivariate analysis, myasthenia gravis was revealed as the only independent prognostic factor for recurrence-free survival (*P* = 0.039). In patients undergoing complete resection for thymic carcinoma, with whom myasthenia gravis was rarely associated, the recurrence-free survival was significantly associated with Masaoka staging and innominate vein or SVC invasion in univariate analysis (*P* = 0.045 and 0.005, respectively; Table 5). Innominate vein or SVC invasion by thymic carcinoma was the only independent factor for recurrence-free survival in multivariate analysis (*P* = 0.012).

**TABLE 1:**
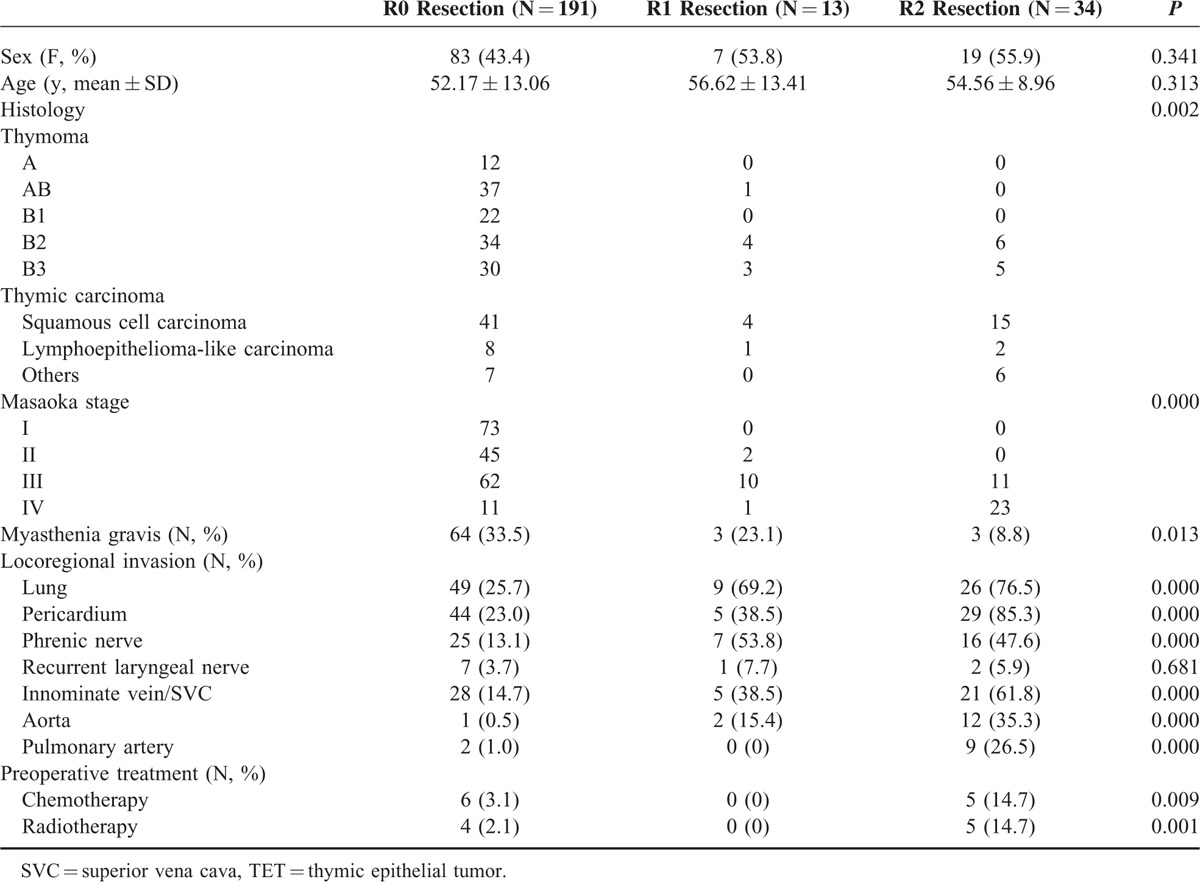
Characteristics of Patients With TETs Undergoing R0, R1, and R2 Resection

**TABLE 2:**
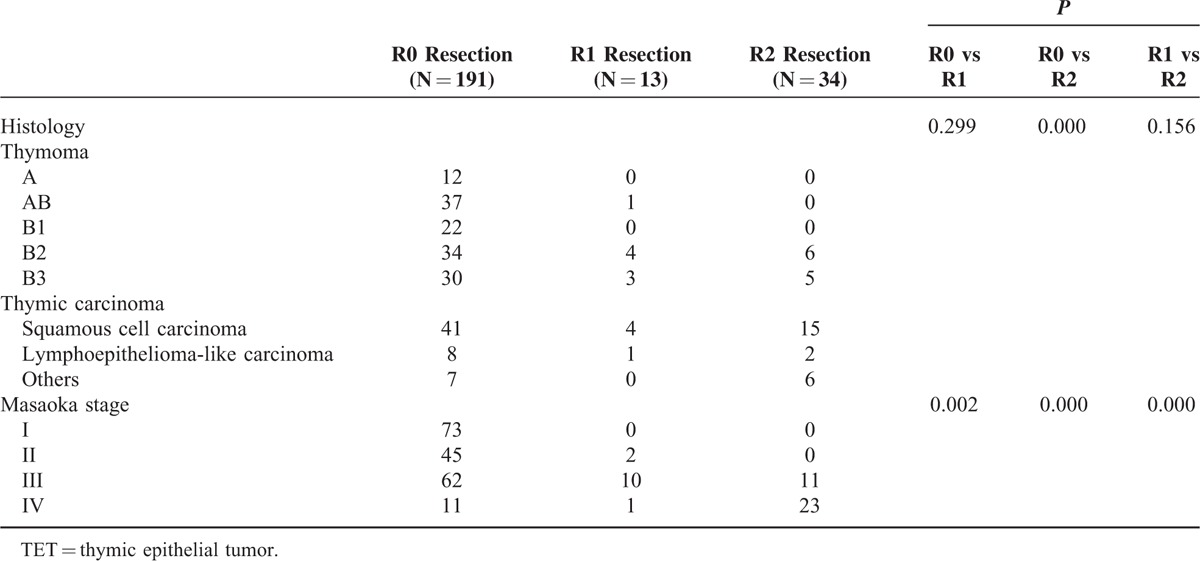
Comparison in Histology and Masaoka Stage Between Patients Undergoing R0, R1, and R2 Resection for TETs

**TABLE 3:**
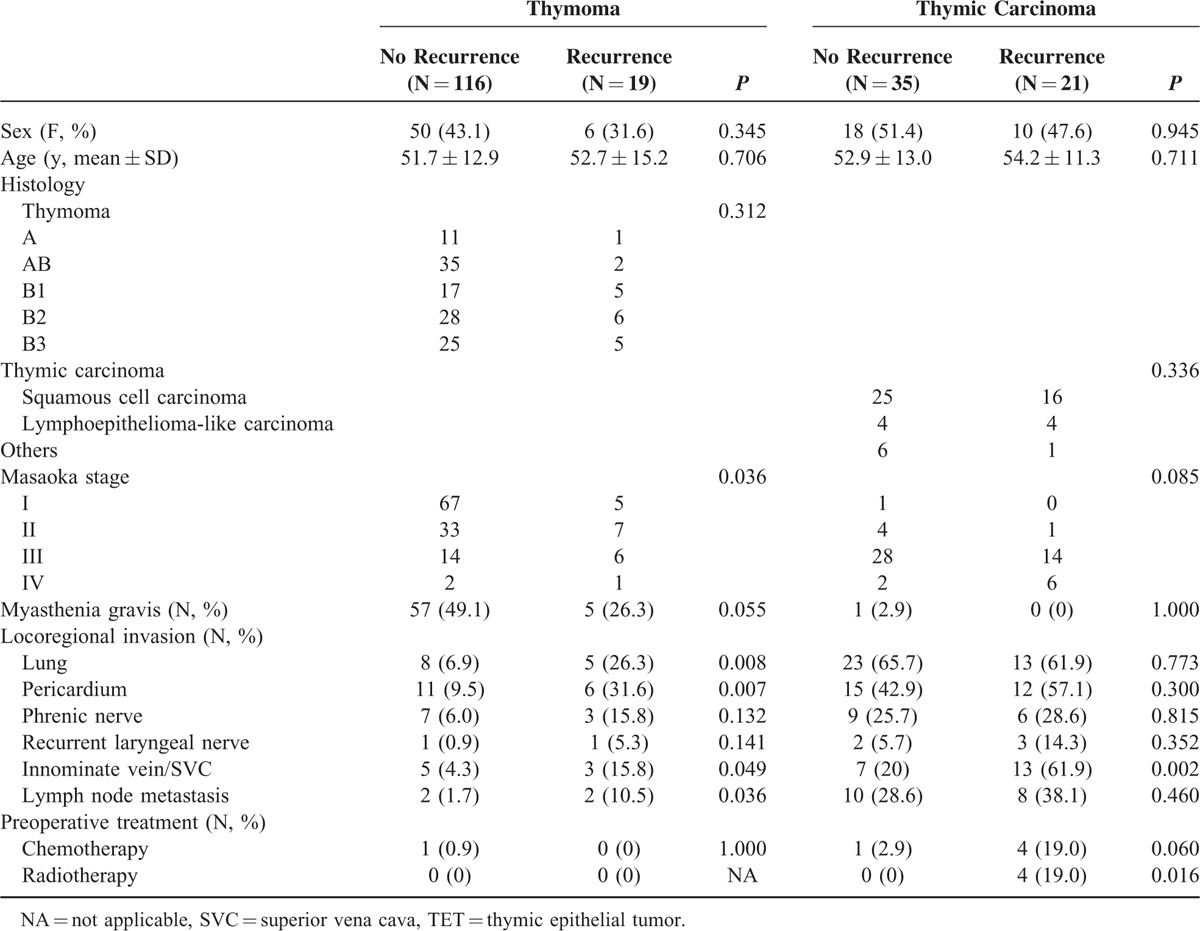
Characteristics of Patients With and Without Disease Recurrence of 191 Completely Resected TETs

**TABLE 4:**
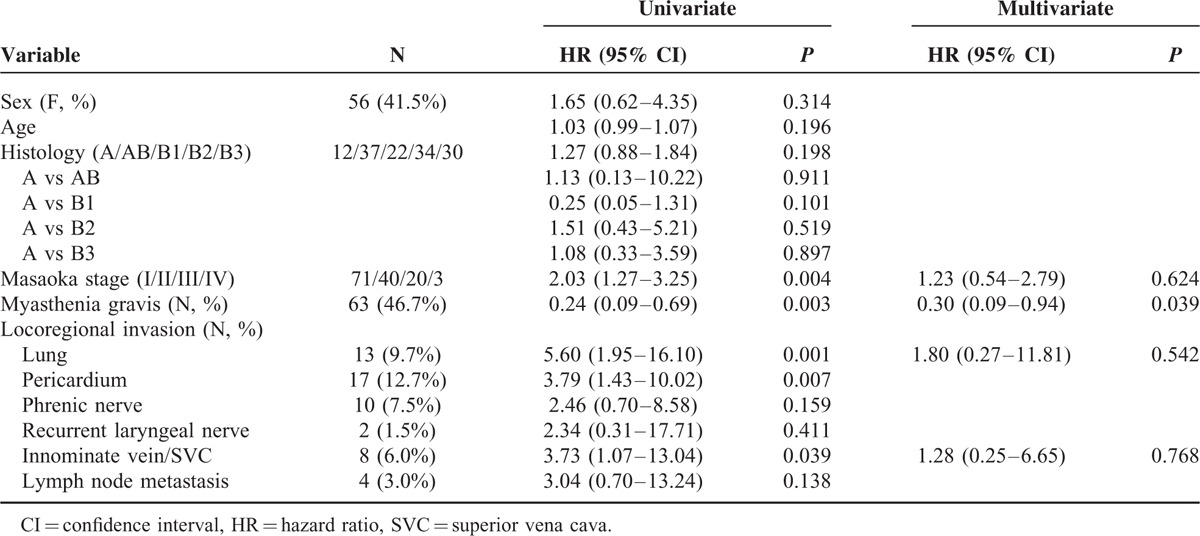
Univariate and Multivariate Analysis of Disease-Free Survival of 135 Patients With Completely Resected Thymoma

**TABLE 5:**
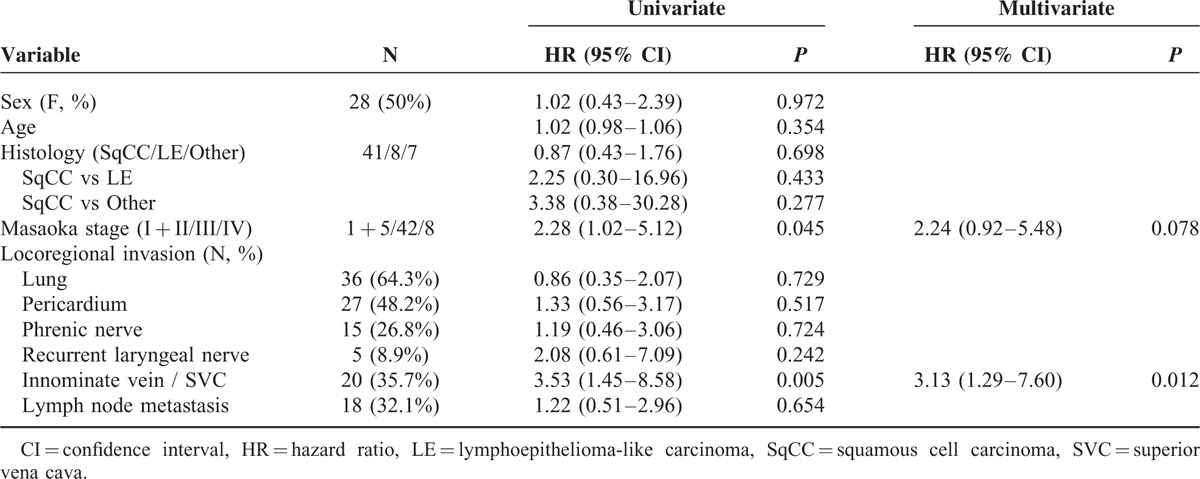
Univariate and Multivariate Analysis of Recurrence-Free Survival of 56 Patients With Completely Resected Thymic Carcinoma

**FIGURE 1:**
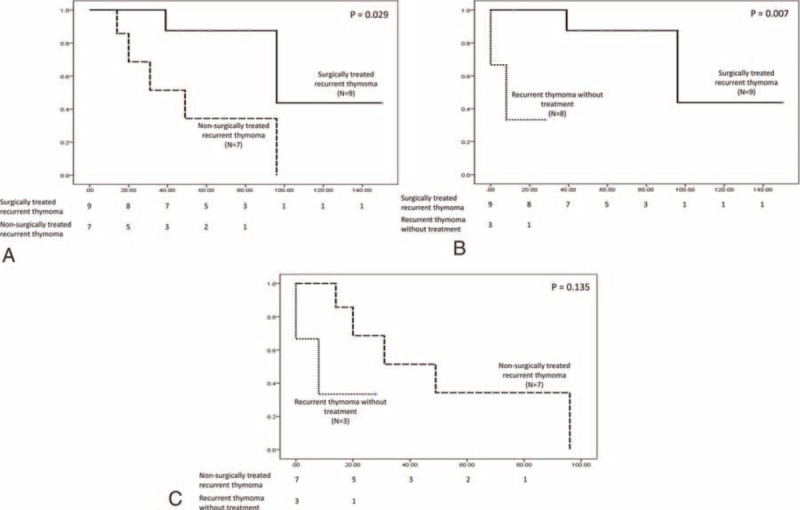
The post-recurrence survival of 19 patients with recurrent thymoma after complete resection was analyzed according to the different treatment modality. Patients who underwent surgical resection followed by chemotherapy had a significantly better post-recurrence survival than those undergoing chemoradiotherapy (A) and those without treatment (B). The post-recurrence survival of those undergoing chemoradiotherapy, however, was not significantly better than those without treatment (C).

**FIGURE 2:**
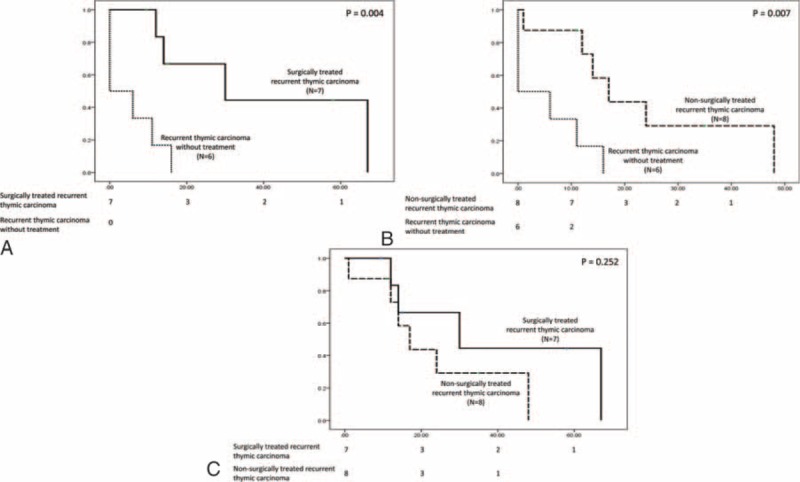
The post-recurrence survival of 21 patients with recurrent thymic carcinoma after complete resection was analyzed according to the different treatment modalities. Patients who underwent surgical resection followed by chemotherapy had a significantly better post-recurrence survival than those without treatment (A). The post-recurrence survival of patients undergoing chemoradiotherapy was also significantly better than those without treatment (B). There was no significant difference in post-recurrence survival between patients undergoing surgery followed by chemotherapy and those undergoing chemoradiation for recurrent thymic carcinoma (C).

There were 2 local, 15 regional, and 12 distal recurrences in the 19 patients with recurrent diseases after complete resection for thymoma (Table 6). Eleven of the 12 distal recurrences occurred in the pulmonary parenchyma, and 1occurred in the chest wall. Nine of these 19 patients underwent surgery followed by chemotherapy for the recurrent disease, whereas 7 were treated with chemoradiotherapy. Three of the 9 patients undergoing resection for recurrent diseases had surgeries for re-recurrences. Among the 21 patients with completely resected thymic carcinoma, there were 4 local, 10 regional, and 18 distant recurrences. Seven patients underwent surgical resection followed by chemotherapy for recurrent diseases. Two of them had repeated surgery for recurrences, and 8 patients were treated with chemoradiotherapy. Nonetheless, 3 patients with recurrent thymoma and 6 patients with recurrent thymic carcinoma did not receive further treatment. The post-recurrence survival of 40 patients with recurrent diseases was further analyzed to evaluate the efficacy of different treatment modalities. In patients with completely resected thymoma, those treated with surgery followed by chemotherapy for recurrent disease had a significantly better post-recurrence survival than those undergoing chemoradiotherapy (*P* = 0.029, Figure 1A), and those without treatment (*P* = 0.007, Figure 1B). Although patients undergoing chemoradiotherapy seemed to have a better post-recurrence survival than those without treatment, the difference was not statistically significant (*P* = 0.135, Fig. 1C). In patients with completely resected thymic carcinoma, those treated with surgical resection followed by chemotherapy for recurrent disease had a significantly better post-recurrence survival than those without treatment (*P* = 0.004, Figure 2A), and so did those undergoing chemoradiation therapy (*P* = 0.007, Figure 2B). The difference of post-recurrence survival between patients undergoing surgery and those undergoing chemoradiotherapy for recurrent thymic carcinoma, however, was not significant (*P* = 0.252, Figure 2C).

**TABLE 6:**
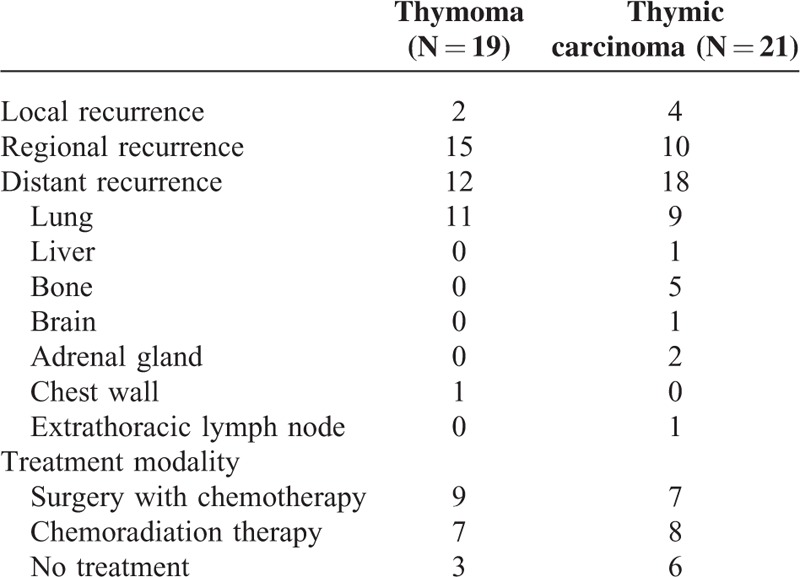
Site and Treatment for Patients With Recurrent Thymoma and Thymic Carcinoma

There were 28 deaths in patients with completely resected thymoma, and 26 in patients with completely resected thymic carcinoma. In the former cohort, there were 8 thymoma-related deaths, 4 non-thymic tumor-related deaths, 1 death due to radiation-induced pulmonary hemorrhage, 9 deaths associated with myasthenic crisis and respiratory failure, 2 cardiovascular deaths, 3 deaths due to bowel ischemia and perforation, and 1 death because of bariatric surgical complications. Among those non-tumor-related deaths in patients with completely resected thymoma, only 1 patient had recurrent disease. Those who died of secondary malignancy including hepatoma, esophageal cancer, gastric cancer, and nasopharyngeal cancer were free of recurrent thymoma.

Twenty-six patient deaths were documented in the cohort of completely resected thymic carcinoma, including 18 cancer-related deaths, 3 non-thymic cancer–related deaths, 1 treatment-related death, and 4 non-cancer-related deaths. Two patients died of lung cancer and 1 died of hepatoma, and they were disease free of thymic carcinoma. One patient died of radiation-induced myocarditis and subsequent heart failure 20 months after resection. Among those 4 non-cancer-related deaths, 1 patient, who was free of thymic carcinoma, died of cerebrovascular disease and central failure. One patient who had surgery for adrenal gland recurrence died of perforated peptic ulcer because of multiple organ failure 4 months after the adrenalectomy. Deep neck infection occurred in 1 patient and resulted in death 1 month after complete resection for thymic carcinoma. The fourth patient died of respiratory failure because of acute exacerbation of chronic obstructive pulmonary disease, aggravated by preexisting radiation pneumonitis. This patient remained free of thymic carcinoma for 168 months.

## DISCUSSION

In light of factors predicting recurrence-free survival in completely resected TETs, our study revealed that instead of Masaoka staging, myasthenia gravis was the positive independent prognosticator for thymoma, while innominate vein or SVC invasion was the negative independent prognosticator for thymic carcinoma. Although the prognosticators and surgical outcomes of TETs have been reported in the literature,^8,13–16^ factors predicting recurrence in patients with thymoma and thymic carcinoma used to be analyzed together, including patients undergoing incomplete resection.^2,3,5–7,16^ Patients of early-stage TET die of other causes, and may also live for many years despite a recurrence. Furthermore, the cause of death is also a suboptimal measure which is affected by other factors, and death is best not mixed together with recurrence end point.^9^ Recurrence-free survival is therefore recommended as the best measure for patients who have successfully undergone complete (R0) resection.^9^ This study enrolled a substantial number of patients undergoing complete resection for thymoma and thymic carcinoma in a 25-year span of a single institution and elucidated the prognostic factor for recurrence-free survival as well as the survival after recurrence. Although patients undergoing complete resection can experience long-term survival,^14,17,18^ recurrence is common in advanced disease, and often earlier and more distant, with lower progression-free survival especially in patients with thymic carcinoma.^5,19,20^ Nonetheless, the introduction of surgery provided not only the opportunity of better recurrence-free survival, but also survival benefit to patients with localized recurrent disease.

Thymoma is usually comorbid with myasthenia gravis, which has been regarded as a positive prognostic factor for overall survival in TETs.^21,22^ On the other hand, the rare occurrence of myasthenia gravis with thymic carcinoma portends its invasiveness before diagnosis. In multivariate analysis, myasthenia gravis was identified as the positive prognosticator for recurrence-free survival in patients with completely resected thymoma, despite the fact that Masaoka staging have been reported to predict recurrence in TETs,^3,7^ which appeared to be one of the prognosticators in univariate analysis. The presence of myasthenia gravis enables timely diagnosis and complete resection for thymoma. In fact, most of the patients undergoing complete resection in our series had stage I and II disease, and the majority of those with myasthenia gravis remained free of recurrence. When it comes to locally advanced disease, the extent of locoregional invasion should be taken into account as demonstrated in univariate analysis for recurrence-free survival. Notably, invasion into the lung but not the pericardium or innominate vein or SVC posed the greatest risk of recurrence in univariate analysis. With the slowly growing nature of thymoma, although the tumor directly invading the lung parenchyma could be readily resected as pericardium or innominate vein or SVC invasion, the abundant lymphovascular structure in the lung parenchyma portended the potential of pleural seeding as well as hematogenous spread. Nonetheless, before locoregional invasion emerged, the presence of myasthenia gravis provided the opportunity of complete resection and long-term recurrence-free survival.

Contradictory results have also been reported whether Masaoka staging predicts the surgical outcome of thymic carcinoma.^23,24^ Our results demonstrated that Masaoka staging was significantly associated with the recurrence-free survival of completely resected thymic carcinoma in univariate but not multivariate analysis. In fact, the number of patients with stage I and II thymic carcinoma was limited and outweighed by that of patients with stage III and IV because of its natural course as an indolent disease. Delineation of locoregional invasion would facilitate our understanding of the tumor behavior, the impact on tumor recurrence and survival, and tailoring the adjuvant therapy in addition to surgical resection. Because of the rare coexistence with thymic carcinoma, myasthenia gravis was not taken into consideration as a prognosticator for recurrence. In patients with completely resected thymic carcinoma, innominate vein or SVC invasion was the only factor predicting recurrence-free survival in multivariate analysis. It is a common belief that invasion of aorta or pulmonary vessels by TETs precludes complete resection because of extensive encasement. Invasion of innominate vein or SVC did not preclude surgery, and long-term survival could be anticipated if the tumor was radically resected as tumor invading the lung or pericardium. Tumor biology also contributes to the differences of recurrence-free survival in thymoma and thymic carcinoma. As thymoma is considered locally slowly growing more than hematogenously spreading, tumor direct invasion into the lung might predispose to local or regional recurrence, whereas distant metastasis is commonly seen in thymic carcinoma at the time of diagnosis. Invasion of the innominate vein or SVC and the potential microscopic metastasis therefore pose a greater risk of recurrence than lung or pericardial invasion in patients with completely resected thymic carcinoma.

The patterns and incidence of recurrence have been reported to vary significantly between thymoma and thymic carcinoma,^3,5,7^ yet patients undergoing incomplete resection were included in the study population. We noticed that recurrences tended to be localized or regional and seldom spread to organs other than lung in completely resected thymoma, whereas distant recurrences were often the cases in completely resected thymic carcinoma. This phenomenon could be attributed to the slowly growing nature yet invisible pleural, parenchymal, or pericardial seeding of thymoma invading the lung or pericardium, and the occult metastasis arising from penetration and spread of thymic carcinoma invading the innominate vein or SVC. The administration of preoperative chemoradiation in our series did not seem to reduce the likelihood of recurrence. As a matter of fact, preoperative chemoradiotherapy was intended to reduce the extent of tumor invasion when vascular invasion precluded direct and complete resection. Even though complete resection could be achieved after neoadjuvant therapy, tumor invasion into SVC was often the case. This may explain that occult metastasis, which occurred mostly along with great vessel invasion, accounts for the recurrent disease after complete resection for thymic carcinoma. The administration of adjuvant chemotherapy after surgical resection should therefore be taken into account for SVC-invading thymic carcinoma.

The tumor histology has been identified as one of the prognosticators of recurrent TETs.^2,3,7^ In most of the studies, thymic carcinoma was categorized and analyzed along with thymoma, and few study focused on the association of tumor relapse with subtyping of thymic carcinoma.^23^ It is reported that the surgical outcome of patients with TETs depends on histologic classification and grade.^3,7,23^ We demonstrated that histologic classification and subtyping did not affect recurrence-free survival in patients with completely resected thymoma or thymic carcinoma. Despite that neuroendocrine and undifferentiated carcinomas were classified as intermediate- and high-grade thymic carcinomas, respectively,^23^ only 1 patient in our series had recurrent disease in this category. This observation implies that the tumor behavior might be different between recurrent and progressive disease, and complete resection provides the only opportunity of long-term survival whatever the tumor histology is.

Surgical resection has been reported to contribute to better survival in patients with recurrent TET.^6,25^ Although surgical resection has been advocated as the preferred treatment modality for recurrent thymoma, it has not been validated on thymic carcinoma. Our results demonstrated that surgical resection followed by chemotherapy provided a better post-recurrence survival than chemoradiotherapy in patients with recurrent thymoma but not in patients with thymic carcinoma. In fact, the optimal treatment for recurrent disease relies on not only the lesion site, but the patient's performance status. Since recurrences in patients with completely resected thymoma were mostly localized, regional, and intrapulmonary lesions, we observed that patients with recurrent thymoma treated with surgery had a better post-recurrence survival than those treated with chemoradiotherapy and those without treatment. With the more rapidly growing nature and malignant tumor biology of thymic carcinoma, it is likely that surgical resection, if not combined with chemotherapy, provided limited benefit in tumor eradication, and that patients with recurrent thymic carcinoma might not be fit enough to undergo surgical resection followed by chemotherapy. Interestingly, recurrence in pleural space and lung parenchyma was the most common in our series, different from some published literature that disease relapse tended to be distant in patients with thymic carcinoma.^5^ We believe this is because our population includes patients undergoing complete resection for thymic carcinoma, whose recurrent diseases result from occult metastasis instead of residual or an existing tumor. Incompletely resected thymic carcinoma, on the contrary, contributes to the earlier and more distant relapse with an evident tumor burden rather than circulating tumor cells. Surgical resection, in our experiences, could be performed not only for the recurrent disease localized in the thorax, but also for a single solitary extrathoracic metastatic lesion. One patient experienced repeated surgical resection for his recurrence in adrenal gland and then retroperitoneum, and achieved long-term survival. Of note, patients who underwent surgical resection for recurrent disease were administered chemotherapy, which might play some role for the post-recurrence survival by eliminating the circulating tumor cells. For those whose recurrent disease is not amenable to surgery, alternative modalities as chemoradiotherapy should be attempted since they might still provide survival benefit.

This study has certain limitations because of its retrospective nature. Even though the number of patients undergoing complete resection was substantial, a study of larger scale is mandatory to further validate our conclusion. Selection bias could also exist since surgery for recurrent diseases was mostly reserved for the physically fit patients. Besides, patients who were amenable of surgical intervention might have an intuitively better survival because the disease extent was limited. Patients who died of causes other than thymic carcinoma remained free of recurrent disease, which might contribute to a shorter overall survival. More research into the impact of adjuvant chemotherapy on the prevention of recurrence is necessary so that the effect of occult metastasis from vascular invasion can be defined.

In conclusion, although Masaoka staging predicts the overall survival of TETs, our study revealed that myasthenia gravis was the independent prognostic factor for recurrence-free survival for completely resected thymoma. In patients with completely resected thymic carcinoma, innominate vein or SVC invasion was significantly associated with recurrence-free survival. In patients with recurrent thymoma, surgical resection should be attempted whenever feasible especially for those with localized disease since it provided benefit for post-recurrence survival. For patients with thymic carcinoma, surgery for recurrence could be considered since it provided benefit for post-recurrence survival as chemoradiotherapy did.
